# Imagining Events Alternative to the Present Can Attenuate Delay Discounting

**DOI:** 10.3389/fnbeh.2019.00269

**Published:** 2019-12-13

**Authors:** Elisa Ciaramelli, Manuela Sellitto, Giulia Tosarelli, Giuseppe di Pellegrino

**Affiliations:** ^1^Department of Psychology, University of Bologna, Bologna, Italy; ^2^The Centre for Studies and Research in Cognitive Neuroscience, University of Bologna, Cesena, Italy; ^3^Department of Comparative Psychology, Institute of Experimental Psychology, Heinrich-Heine University of Düsseldorf, Düsseldorf, Germany

**Keywords:** delay discounting, episodic future thinking, imagination, intertemporal choice, mental time travel

## Abstract

Previous studies have shown that delay discounting (DD), the tendency to prefer smaller-immediate to larger-delayed rewards, decreases following vivid imagination of future events. Here, we test the hypothesis that imagining complex events alternative to direct (perceptual) experience, whether located in the future, the past, or even the present, would reduce DD. Participants (*N* = 250) imagined future events (Future condition), remembered past events (Past condition), imagined present events (Present-imagine condition), or reported on the current events (Present-attend condition), and then made a series of intertemporal choices about money and food. Compared to attending to the present, imagining the future reduced DD, but this only held for individuals who claimed vivid pre-experiencing of future events. Importantly, a similar attenuation of DD was found in the Past and Present-imagine conditions, suggesting that a shift in perspective from the perceptual present towards mentally constructed experience can downplay the appraisal of immediate rewards in favor of larger-delayed rewards, regardless of the location of the imagined experience in subjective time.

## Introduction

Intertemporal choice requires trading off between options attainable at different times. One example is deciding whether to spend the afternoon rambling or instead stay home and do work in view of a future exam. Individuals tend to prefer immediate rewards to long-term rewards of larger value. Formally, this phenomenon reflects the decrease in subjective value of a reward as the delay until its receipt increases, known as delay discounting (DD; Frederick et al., 2002; Sellitto et al., 2011). The rate at which future rewards are discounted (DD rate) varies widely across individuals (Soman et al., 2005; Peters and Büchel, 2011) and is particularly high in clinical conditions characterized by impulsive and shortsighted behavior, for example, addicted subjects (Kirby and Petry, 2004; Bulley and Gullo, 2017), compulsive gamblers (Holt et al., 2003), obese individuals (Amlung et al., 2016), and patients with lesions to the ventromedial prefrontal cortex (vmPFC; Sellitto et al., 2010; Peters and D’Esposito, 2016). Indeed, individuals with high vs. low impulsivity show greater difference between immediate and delayed reward outcomes in the reward positivity, an ERP component reflecting reward-related signals in the anterior cingulate cortex (ACC; Schmidt et al., 2016). Importantly, DD rates have been found to vary greatly also *within* individuals (Peters and Büchel, 2011; Lempert and Phelps, 2016), depending on contextual variables and ongoing cognitions (Berns et al., 2007), which is relevant for understanding the component processes governing DD, as well as conceiving “cognitive tools” to contrast it.

Recent research has shown that episodic future thinking (Suddendorf and Corballis, 2007; Schacter et al., 2007, 2017; D’Argembeau et al., 2010), the mental simulation of events relevant to one’s own future, reduces DD. Peters and Büchel (2010) compared a standard DD task with a modified task in which personal future events imagined at given delays were provided as cues during intertemporal choices involving rewards available at those delays. In the episodic cue (compared to the standard) task, individuals’ preferences shifted towards future rewards, and the reduction of DD rates was associated with the vividness of the imagined future event and with increased functional coupling between the hippocampus and vmPFC and ACC regions associated with reward processing and valuation (Kable and Glimcher, 2007; Peters and Büchel, 2010; Benoit et al., 2011). The effect of episodic cueing on DD is consistently found in healthy individuals (Benoit et al., 2011; Liu et al., 2013; Lin and Epstein, 2014; Bromberg et al., 2017; O’Donnell et al., 2017, 2018; Zhang et al., 2018; Bulley et al., 2019), as well as patients with substance abuse disorders (Daniel et al., 2013; Snider et al., 2016), in whom it extends to real-world indices of impulsive choice, such as impulsive drinking or eating (Daniel et al., 2013; Dassen et al., 2016; see also Wu et al., 2017). In contrast, consistent with Peters and Büchel’s (2010) finding that episodic cueing effects on DD are conditional upon the imagination of vivid future events, no episodic cueing effect has been observed in amnesic patients with hippocampal damage (Palombo et al., 2014; but see Kwan et al., 2015), who cannot construct detail-rich future events (Race et al., 2011; see also De Luca et al., 2018) to use for decisions.

How does episodic future thinking promote future-oriented choice? One view is that the vivid imagination of future events triggers emotions in the here-and-now (Gilbert and Wilson, 2007; Damasio, 2009), rendering future rewards more emotionally engaging and desirable, and therefore capable to compete for salience with rewards that are available immediately (Boyer, 2008; Ciaramelli and di Pellegrino, 2011; Cole and Berntsen, 2016). There is some evidence, indeed, that positive but not negative episodic future thinking reduces DD (Liu et al., 2013; Zhang et al., 2018), although other research using large samples has detected episodic cueing effects even following neutral (Lin and Epstein, 2014) and even negative future thinking (Bulley et al., 2019). Episodic future thinking may also alter participants’ time perspective (Lin and Epstein, 2014), increase personal connectedness to the future (O’Donnell et al., 2017), and promote a more concrete and detailed construal of future events (Cheng et al., 2012; Lebreton et al., 2013), biasing choice accordingly.

Although most studies so far have investigated the effect of episodic *future* thinking on DD (e.g., Peters and Büchel, 2010; Benoit et al., 2011; Liu et al., 2013; Lin and Epstein, 2014), self-projection into the future correspondingly entails a detachment from direct (perceptual) experience, and it is possible that this inherent component of mental time travel is capable, in itself, to attenuate DD, at least in part. If so, imagining any event alternative to the present experience should reduce DD. There is initial evidence that this is the case. Lempert et al. (2017) found that autobiographical memory retrieval reduced DD, suggesting that projecting oneself into the past also helps overcome the bias towards immediate gratification (see also Ersner-Hershfield et al., 2009; Mitchell et al., 2011).

Despite this initial evidence, it is still unclear whether reducing DD *via* self-projection would necessarily require the imagination of events located in the future, or rather, imagining any event removed from the perceptual present would suffice. Consistent with the latter possibility, overlapping brain regions are engaged while individuals mentally project themselves in situations alternative to the present, be these located in the future, located in the past, alternative versions of the past (e.g., counterfactual thinking), or even atemporal (Addis et al., 2007, 2009; Hassabis et al., 2007; De Brigard et al., 2013; Benoit and Schacter, 2015). Also, DD was found negatively associated with mind-wandering, such that individuals prone to shifts of attention away from current tasks/events towards inner thoughts (e.g., memories, plans; Smallwood et al., 2011) are also those more capable to wait for larger-later rewards (Smallwood et al., 2013), and both mind-wandering and patient intertemporal choice relate to gray matter volume in vmPFC (Bernhardt et al., 2014) and are hindered by vmPFC damage (Sellitto et al., 2010; Bertossi and Ciaramelli, 2016).

The first aim of the present study is to test whether imagining the future, remembering the past, and imagining an alternative present, as all instances of self-projection away from perceptual towards mentally constructed experience, are (equally) effective in reducing DD compared to maintaining attention on the present. This would contribute to specify the component processes underlying episodic cueing effects on DD and to reveal viable alternatives to reduce DD. The second aim of the study pertains to degree of retrieval support and structure characterizing episodic cueing of intertemporal choice. In most studies, individuals first imagine future experiences, and then tags reminding of these experiences are embedded in single trials of the DD task. This heavily structured cueing is highly effective in reducing DD, but may be difficult to adapt flexibly to clinical practice demands and, even more so, to adopt spontaneously in daily life. In fact, individuals often resort to extemporaneous “metacognitive tools” to exert control over disadvantageous mental processes. For example, we “count to ten” before we act when angry, and thoughts about the future are reportedly used in daily life to direct action (D’Argembeau et al., 2011). Thus, here we ask whether imagining events removed from the perceptual present before (with no cue during) intertemporal choice would make choices less present-oriented and more farsighted, reducing DD.

To these aims, four participant groups imagined future events, remembered past events, imagined alternative present events, or described the current events, and then underwent standard DD tasks involving monetary or food rewards. We expect to replicate that self-projection into the future would reduce DD compared to attending to the present, and that the episodic cue effect on DD would depend on the vividness of future thinking (Peters and Büchel, 2010; Palombo et al., 2014). If the effect of episodic future thinking on DD is mediated, at least in part, by the detachment from the present inherent to future thinking, then a reduction of DD should be observed also when participants remembered the past or imagined an alternative present.

## Materials and Methods

### Participants

Participants in the study were 250 healthy individuals (mean age: 35.60 years, range: 19–75; mean education: 15.24, range: 5–23) recruited at the Bologna and Cesena campuses of the University of Bologna, who gave informed consent to participate according to the Declaration of Helsinki (International Committee of Medical Journal Editors, 1991) and the Bioethical Committee of the University of Bologna. Participants were randomly assigned to four different groups, namely, the Future group (*N* = 59), the Past group (*N* = 56), the Present-imagine group (*N* = 72), and the Present-attend group (*N* = 63), which did not differ in gender distribution, age, education level, body mass index (BMI; Aiello et al., 2018), and the order of administration of the two intertemporal choice tasks described below (*H* < 1.23, *p* > 0.57 in all cases; see Table 1 for the groups’ characteristics).

**Table 1:** Participant groups’ characteristics.

Group	F:M	Mean age (years)	Mean education (years)	BMI
Present-attend	32:31	35.4 (13.0)	15.0 (2.4)	23.0 (3.2)
Past	31:25	36.2 (13.6)	15.1 (2.8)	23.5 (3.2)
Future	33:26	35.4 (13.6)	15.2 (3.2)	23.6 (4.2)
Present-imagine	43:29	35.4 (13.6)	15.6 (3.1)	23.2 (3.7)

*Notes. F, females; M, males; BMI, body mass index. Numbers in parenthesis are SDs*.

### Time Tasks

Before the intertemporal choice tasks, participants either attended to the present or re-/pre-/experienced an event alternative to the current experience. Participants in the Present-attend group were required to focus on their current experience. They had to list on a paper sheet what was on their desk, then look around and describe in as much detail as they could the environment they were immersed in and what was happening at that moment. Participants in the Past group were required to remember, one at a time, two specific events from their past as vividly as they could (i.e., trying to re-experience the events while recalling them): the first occurred about 1 year before, and the second occurred about 3 years before. They then described the events briefly on a paper sheet. Participants in the Future group were required to imagine, one at a time, two events that might happen to them in the future as vividly as they could (i.e., trying to pre-experience the events while imagining them): the first to occur in about 1 year and the second in about 3 years. They then described the events briefly on a paper sheet. Lastly, participants in the Present-imagine group imagined and then described, one at a time, two events that might happen to them in the present, different from the one they were currently experiencing, but that could be located in the same spatial context and involve the same people/objects. The emotional content (positive vs. negative) of (re)constructed experience was not the focus of our experimental question/manipulation. Nevertheless, to promote the vivid simulation of events alternative to the perceptual present, we encouraged participants to remember or imagine something positive or neutral in nature, which they would feel comfortable re-/pre-/experiencing in detail.

Across experimental conditions, after having described/remembered/imagined each event, participants rated, on a 5-point Likert scale (ranging from 1 = “a lot,” to 5 = “not at all”), the degree to which they felt that: (1) describing/remembering/imagining that event was easy (difficulty scale); (2) describing/remembering/imagining that event elicited emotions (emotion scale); and (3) they were attentive to the present (in the case of the Present-attend group) or vividly re-/pre-/experiencing an alternative (past/future/present) event (in the case of the Past/Future/Present-imagine groups; vividness scale).

### Intertemporal Choice Tasks

Immediately after the time task, participants underwent two intertemporal choice tasks measuring DD for two different types of hypothetical reward, namely, food and money, previously described in Sellitto et al. (2010). We used chocolate bars as the food reward after assuring all participants liked and could eat chocolate. In each computerized task, subject chose between an amount of reward that could be received “now” (smaller-immediate reward) and an amount of reward that could be received after a specific delay (larger-later reward; Sellitto et al., 2010). Participants made five choices at each of six delays of availability of the larger-later option: 2 days, 2 weeks, 1 month, 3 months, 6 months, and 1 year. The order of blocks of choices pertaining to different delays was randomly determined across participants. Within each block of five choices, the delayed amount was fixed at 40 units (40€, 40 chocolate bars), whereas the amount of the immediate reward was adjusted based on the participant’s choices using a staircase procedure that converged on the amount of the immediate reward that was equal, in subjective value, to the delayed reward (Sellitto et al., 2010; Sellitto and di Pellegrino, 2016). In each block, the participant always chose between a delayed amount of 40 units and an immediate amount of 20 units. If the participant chose the immediate reward, the amount of the immediate reward decreased in the following trial; if the participant chose the delayed reward, the amount of the immediate reward increased in the following trial. The adjustment size on the immediate reward decreased with successive choices: the first adjustment was half of the difference between the immediate and the delayed reward, whereas it was half of the previous adjustment for later choices (Myerson et al., 2001). This procedure ended when the subject had made five choices at one specific block (delay), after which a new series of choices at another delay began. For each trial in a block, the immediate amount that would have been presented on the sixth trial of a delay block was taken as the estimate of the “indifference point” between smaller-immediate and larger-later rewards, thus representing the subjective value of the delayed reward at that delay.

We assessed DD rates estimating the area under the curve (AUC; Myerson et al., 2001; Sellitto et al., 2010; Peters and D’Esposito, 2016). Delays and subjective values were first normalized. Delays were expressed as a proportion of the maximum delay (360 days), and subjective values were expressed as a proportion of the delayed amount (40 units). Delays and subjective values were then plotted as *x* and *y* coordinates, respectively, to construct a discounting curve. Vertical lines were drawn from each *x* value to the curve, subdividing the AUC into a series of trapezoids. The area of each trapezoid was calculated as (*x*_2_ − *x*_1_)(*y*_1_ + *y*_2_)/2, where *x*_1_ and *x*_2_ are successive delays, and *y*_1_ and *y*_2_ are the subjective values associated with these delays. The AUC is the sum of the areas of all the trapezoids. The AUC varies between 0 and 1. The smaller the AUC, the steeper DD, the more participants were inclined to choose small-immediate rewards over larger-delayed rewards.

### Procedure

Upon arrival, participants filled in a demographics questionnaire, then performed one of the time tasks, and finally underwent the food and money DD tasks, administered in a counterbalanced order.

### Statistical Analyses

All our variables (Kolmogorov-Smirnov *d* > 0.08, *p* < 0.01), with the exception of the AUC for money (*d* = 0.06, *p* > 0.20), were non-normally distributed, and therefore, we analyzed our data mainly resorting to non-parametric statistics. Between-group differences were assessed with Kruskal–Wallis ANOVAs and Mann–Whitney *U* tests, within-group differences with Wilcoxon matched-pairs tests and correlations with the Spearman test. To provide as informative an analysis of participants’ performance as possible, data on the AUC for money, normally distributed, were additionally analyzed using parametric ANOVA. Unless otherwise noted, we report effects significant at *p* < 0.05, two-tailed.

## Results

### Whole Sample

#### Subjective Ratings

We first compared the self-reported levels of difficulty, emotion, and vividness associated with attending to the present vs. an alternative past/present/future event by running Kruskal–Wallis ANOVAs on individuals’ subjective ratings (see Table 2). We found group differences in difficulty (*H* = 27.35, *p* < 0.0001), such that participants found imagining future events, imagining present events, and remembering past events more difficult than describing the present (*Z* > 3.66, *p* < 0.001 in all cases), whereas remembering and imagining events were rated as comparably difficult (*p* > 0.75 in all cases). There were also significant differences in emotion ratings (*H* = 109.04; *p* < 0.0001): remembering the past (*Z* = −2.40, *p* = 0.02) and imagining the future (*Z* = −4.55, *p* = 0.0005) elicited more emotions than imagining an alternative present, which in turn elicited more emotions than describing the present (*Z* = 7.57, *p* = 0.00001). Finally, participants reported experiencing the present or alternative past, present, and future events with comparable levels of vividness (*p* = 0.08).

**Table 2:** Subjective ratings in the time task.

Group	Mean difficulty	Mean emotion	Mean vividness
Present-attend	1.4 (0.6)	4.3 (0.9)	2.2 (1.1)
Past	2.0 (0.9)	2.4 (1.0)	2.4 (1.0)
Future	2.0 (0.9)	2.1 (0.8)	2.2 (1.0)
Present-imagine	2.0 (0.8)	2.8 (0.8)	2.5 (0.8)

*Notes. Numbers in parenthesis are SDs*.

#### DD

**Figure 1:**
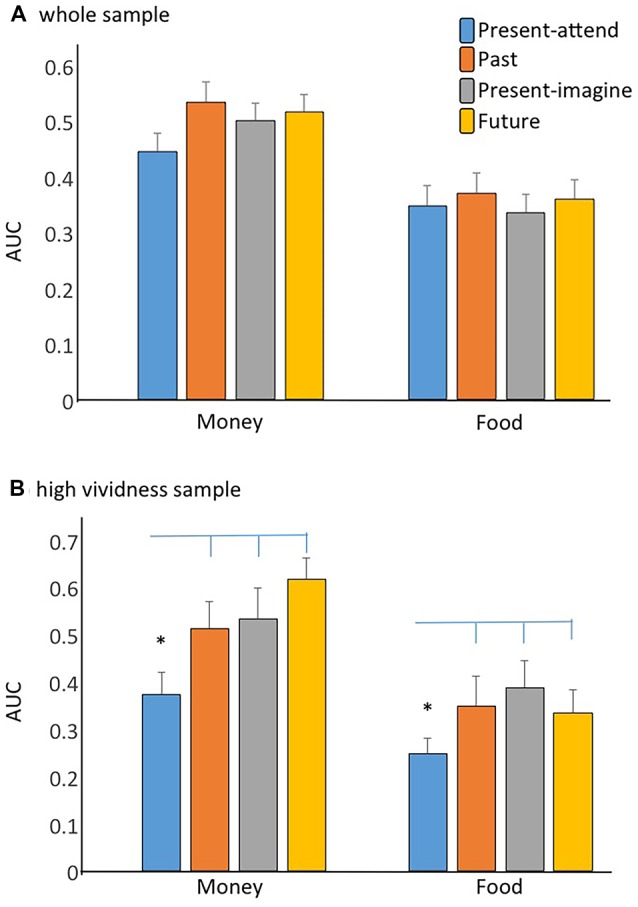
Area under the curve (AUC) for money and food in the Past, Future, Present-attend, and Present-imagine groups of the whole sample **(A)** and of the high vividness sample **(B)**. Error bars indicate the SEM, and asterisks denote significant findings (*p* < 0.05).

Despite differences in the subjective experience associated with the different time conditions, Kruskal–Wallis ANOVAs on AUCs showed no significant group difference for either monetary or food rewards (both *H*s < 1.4, *p*s > 0.27), suggesting that detaching from the present to experience an alternative event does not generally result in a change in DD rates (see Figure 1A). Consistent with previous reports (Odum and Rainaud, 2003; McClure et al., 2007; Sellitto et al., 2010), food rewards were discounted more steeply than money across groups (Wilcoxon *Z* > 3.08; *p* < 0.002 in all cases). An ANOVA on AUCs for money with group as between-subject factor confirmed no effect of group (*F*_(3,246)_ = 1.22; *p* = 0.30).

#### Exploratory Correlations

We ran exploratory Spearman correlation analyses to investigate the relation between AUC rates and demographic and individual variables (age, education, and BMI) and subjective ratings (emotion, vividness, difficulty). The Bonferroni-corrected significance level was *p* < 0.004. The analyses showed that the AUC for money and food did not correlate significantly with age (*p* > 0.38 in both cases), education (*p* > 0.14 in both cases), BMI (*p* > 0.10 in both cases), emotion ratings (*p* > 0.13 in both cases), vividness ratings (*p* > 0.71 in both cases), or difficulty ratings (*p* > 0.02 in both cases).

### High Vividness Subsamples

Based on previous findings that self-projection into the future reduced DD significantly only in participants who reported having imagined future events with high vividness (Peters and Büchel, 2010; Palombo et al., 2014; see also Lin and Epstein, 2014), we reasoned that the lack of cueing effects in this study may relate to participants not having succeeded at re-/pre-/experiencing vividly events alternative to the present in the Past, Future, Present-imagine conditions, or at fully attending to the present in the Present-attend condition, blurring cueing effects on DD. We therefore restricted our analyses to subgroups of participants who reported the highest level of vividness (score = 1) in at least one of the two events they had remembered from the past (Past_v group; *N* = 25), imagined to occur in the future (Future_v group; *N* = 23), imagined to occur in the present (Present-imagine_v group; *N* = 16), and described from the present (Present-attend_v group; *N* = 24). The high vividness subgroups were still matched for gender distribution, age, education level, and BMI (*H* < 5.60, *p* > 0.13 in all cases; see Table 3 for groups’ characteristics). Also, high vividness participants (collapsing across subgroups; *N* = 88) did not differ in age, education, gender balance, or BMI (all *p*s > 0.10) from participants in the whole sample who never reported having experienced alternative past/present/future events vividly or fully attended to the present (*N* = 162).

**Table 3:** High vividness groups’ characteristics.

Subgroup	F:M	Mean age (years)	Mean education (years)	BMI
Present-attend_v	15:10	30.1 (10.2)	15.7 (2.7)	22.5 (2.7)
Past_v	12:11	36.1 (14.9)	14.0 (2.9)	23.4 (3.4)
Future_v	10:12	36.4 (15.5)	13.8 (4.0)	22.6 (3.0)
Present-imagine_v	7:9	36.4 (13.2)	15.6 (2.2)	23.3 (2.7)

*Notes. F, females; M, males; BMI, body mass index. Numbers in parenthesis are SDs*.

#### Subjective Ratings

Kruskal–Wallis ANOVAs on subjective ratings in the high vividness groups (Past_v, Future_v, Present-attend_v, Present-imagine_v), displayed in Table 4, showed group differences in difficulty ratings (*H* = 9.68, *p* = 0.02), such that vividly remembering past events (*Z* = 2.62, *p* = 0.02) and imagining present events (*Z* = 2.82; *p* = 0.01) were perceived as more difficult than attending to present events, whereas differences between imagining the future and attending to the present (*p* = 0.06), or between remembering and imagining (future and present) events were not significant (*p* > 0.45 in all cases). Emotion ratings also differed across groups (*H* = 47.17; *p* = 0.0001), such that participants reported more emotions in association with remembering past events (*Z* = 5.31; *p* < 0.001) and imagining future (*Z* = 5.61; *p* < 0.001) and present events (*Z* = 4.94; *p* < 0.001) than with attending to present events. Imagining future events was associated with similar levels of emotion than remembering past events (*p* = 0.26), but elicited more emotions than imagining present events (*Z* = 2.79; *p* = 0.005). Group differences in vividness were not significant (*p* = 0.065).

**Table 4:** High vividness groups’ ratings in the time task.

Group	Mean difficulty	Mean emotion	Mean vividness
Present-attend_v	1.1 (0.4)	4.6 (0.9)	1.4 (0.6)
Past_v	1.8 (1.2)	2.0 (1.1)	1.6 (1.0)
Future_v	1.5 (0.9)	1.7 (0.7)	1.3 (0.5)
Present-imagine_v	1.7 (0.8)	2.2 (1.2)	1.4 (0.5)

*Notes. Numbers in parenthesis are SDs*.

#### DD

In the high vividness groups, AUCs for money and food were normally distributed (Kolmogorov-Smirnov *d* < 0.10; *p* > 0.20), and therefore, group differences were assessed with parametric tests. An ANOVA on AUCs with group (Past_v, Future_v, Present-attend_v, Present-imagine_v) and reward (food, money) as factors yielded a significant effect of reward (*F*_(1,84)_ = 28.70; *p* < 0.001), such that food was discounted more steeply than money across groups, and a significant effect of group (*F*_(3,84)_ = 3.40; *p* = 0.02). *Post hoc* comparisons, run with the Newman-Keuls tests, showed a reduced DD in participants who vividly remembered past events (*p* = 0.04), imagined future events (*p* = 0.03), and imagined present events (*p* = 0.03) compared to those who focused on the present before making intertemporal choices, while there were no significant differences across the Past_v, Future_v, and Present-imagine_v groups (*p* > 0.62 in all cases; see Figure 1B). We obtain similar findings using non-parametric statistics.

#### Exploratory Correlations

Spearman correlation analyses (Bonferroni-corrected significance level: *p* < 0.004) showed that, in the high vividness groups, the AUC for money and food did not correlate significantly with age (*p* > 0.20 in both cases), education (*p* > 0.54 in both cases), BMI (*p* > 0.81 in both cases), difficulty ratings (*p* > 0.33 in both cases), or vividness ratings (*p* > 0.64 in both cases). However, participants who reported higher levels of emotion in the time task were also those who showed lower DD rates for money (*r*_Spearman_ = −0.33, *p* = 0.001). The correlation between emotion ratings and AUCs for food was not significant (*p* = 0.41).

## Discussion

Previous research has shown that engaging in episodic future thinking during intertemporal choice reduces individuals’ natural disposition towards immediate gratification in favor of choices with long-term benefits, attenuating DD (e.g., Peters and Büchel, 2010; Benoit et al., 2011; Daniel et al., 2013; Lin and Epstein, 2014; Dassen et al., 2016; Bromberg et al., 2017; Bulley and Gullo, 2017; Bulley et al., 2019). In most DD tasks, episodic future thoughts are instilled in intertemporal choice using online cues that point to (previously imagined) experiences to occur at the relevant task delays. Episodic cueing of intertemporal choice results in increased activity in a core network associated with episodic future thinking (Benoit and Schacter, 2015) and in increased functional coupling between the hippocampus and vmPFC and ACC regions that relates directly to individuals’ shifts in preference for future options (Peters and Büchel, 2010). These findings suggest that episodic future thinking up-regulates the salience and utility of future relative to immediate choice options in the valuation system, biasing preference accordingly. Indeed, episodic cueing effects on DD are contingent upon the vividness of episodic imagery (Peters and Büchel, 2010; Palombo et al., 2014).

Two main questions motivated the present study. First, we sought to inquire further on the mechanisms mediating episodic cueing effects on DD by asking whether imagining any event alternative to direct (perceptual) experience, whether located in the future, the past, or even the present, would be able to reduce DD. Second, we tested whether episodic future thinking and other types of event construction would modulate DD even if subjects mentally projected themselves in time before the DD task and had no reminder of their mental time travel during the task. Our results show that imagining future events, remembering past events, and imagining present events before making intertemporal choices are equally associated with reduced DD compared to maintaining attention on the current event. This finding confirms and extends previous evidence that autobiographical memory retrieval reduces DD (Lempert et al., 2017), and that the number of episodic details produced during autobiographical memory retrieval correlates negatively with DD rates (Peters et al., 2017). Notably, this effect was not detected across participants, but focusing on those who self-reported having succeeded fully in the time task, experiencing vividly a mentally constructed past/future/present event as opposed to staying focused on the perceptual present, which were about one-third of the original sample. This finding not only makes contact with the tight relation observed between episodic cueing effects on DD and the quality of episodic simulation (Peters and Büchel, 2010; Palombo et al., 2014; Peters et al., 2017), but also draws attention to the fact that mentally traveling in subjective time is a demanding activity. We will return to this point later.

Our main finding that self-projecting into constructed experience, whether located in the future, past, or present, was associated with reduced DD compared to attending to the perceptual present indicates that the well-documented effect of episodic future thinking on DD is attributable, at least in part, to component processes episodic future thinking shares with other instances of self-projection, as testified by shared neural bases (Addis et al., 2007, 2009; Hassabis and Maguire, 2007; Hassabis et al., 2007; Nyberg et al., 2010; Kurczek et al., 2015; Bertossi et al., 2016). Which component processes of self-projection may underlie the DD decrease? First, imagining the future, imagining an alternative present, and remembering the past all entail a detachment from direct experience and processing of information that is not present to the senses. Activity in several nodes of the core autobiographical network, such as the medial prefrontal cortex (Burgess et al., 2003; Gilbert et al., 2006) and the posterior parietal cortex (Cabeza et al., 2008; Ciaramelli et al., 2008; see also Nyberg et al., 2010), has been conceptualized as mediating the allocation of attention to internal (vs. external) sources of information, a process inherent to memory retrieval. Directing attention away from perceptual reality towards inner, mentally constructed experience may downregulate the appraisal of immediate rewards, reducing the valuation gap normally present between immediate and future rewards, hence DD (Ballard and Knutson, 2009; Smallwood et al., 2011; Macrae et al., 2017). Consistent with this interpretation, mind-wandering, the drift of attention away from external tasks/events towards internally generated information (e.g., thoughts, memories, plans; Smallwood et al., 2011), which is characterized by reduced cortical analysis of external events (Smallwood et al., 2008; Kam et al., 2011), is also associated with low DD rates (Smallwood et al., 2013). In addition, the vivid simulation of future, past, and present events alternative to the current experience likely mobilized construction/elaboration processes that also operate while anticipating what receiving a reward in the future would be like. These processes may have promoted a detailed imagination of future outcomes, which is associated with low DD rates (Hakimi and Hare, 2015; for a discussion see Bar, 2010). Indeed, activity in vmPFC (Mitchell et al., 2011; Cooper et al., 2013) and the hippocampus (Lebreton et al., 2013) while thinking about the future predicts individual DD rates. Moreover, steep DD correlates with impaired mental time travel in vmPFC patients (Bertossi et al., 2016).

It should be noted that participants self-reported more emotion in the Past, Future, and Present-imagine conditions compared to the Present-attend condition. Given that we encouraged the (re)construction of relatively positive events, one may wonder whether reduced DD merely related to positive affect. We do not think this is the case. Had positive affect played a major role in the reduction of DD in the high vividness sample, the same reduction would have been detected in the whole sample, as even the whole sample self-reported more emotion in the Past, Future, and Present-imagine conditions compared to the Present-attend condition. However, there was no modulation of DD across time conditions in that sample. In addition, emotion ratings did not correlate with DD in the whole sample, but only in the high vividness sample. That is, it is not emotion in general that necessarily related to DD, but emotion associated with an actual shift of the self in time, which participants in the high vividness—but not the whole—sample experienced. These findings suggest that constructed experience is most effective in reducing DD when emotionally engaging, in addition to vivid and detailed. In line with our data, in Lempert et al. (2017), DD decreased following autobiographical memory retrieval, but not non-mnemonic (non-personally relevant) imagery. We take emotional adherence to constructed experience as proof of a reliable disengagement from the present and adoption of a different self-perspective, which reinforces our hypothesis of a link between DD and self-projection. Clearly, our data do not allow for specifying which specific component process of self-projection is most closely tight to the DD reduction (e.g., disengagement from the present, detailed simulation of the alternative event, emotional engagement with the event), which remains a topic for future inquiry.

As for the second aim of the study, our results suggest that a significant reduction in DD can be observed even if individuals engage in self-projection before making intertemporal choices, without having any further cue during choice itself. This finding is worth noting as it makes contact with new research aimed at delineating the boundary characteristics within which episodic cueing manipulations are effective in altering DD (O’Donnell et al., 2017, 2018; Hollis-Hansen et al., 2019). For example, in most previous experiments, participants create cues about future events that occur at the time delays of the DD task, which are then represented during the task. Hollis-Hansen et al. (2019) have found recently that DD is reduced even when the episodic future cues do not match the temporal delays of the task, suggesting that the generation of episodic future cues might in itself be enough to increase the valuation of future rewards (see also Stein et al., 2017). Our results are in line with these findings, and also indicate that the constraint to have episodic cues located in the future may also be not necessary, provided that imagined events are vivid enough to have the potential to draw attention away from information present to the senses, towards inner experience.

In summary, we have found that mentally constructing vivid events alternative to perceptual experience is associated with reduced DD rates compared to attending to the perceptual present, no matter the precise (past, future, present) location of constructed experience in subjective time. This finding points to self-projection as an effective, adoptable, and generalizable strategy to protect one’s intertemporal decisions from impulsivity.

We conclude by highlighting the limitations and future directions of this work. One limitation is that we used a between-subject design. Therefore, even though we controlled for a considerable number of demographical and individual variables (age, education, BMI), we cannot exclude that the observed effects are due, at least in part, to group differences in uninvestigated variables, and it will be important to confirm them using within-subject designs. As anticipated, our data indicate that self-projection is not trivial an activity: only one-third of our sample succeeded in assuming the desired time perspective in the time task. Future studies should therefore inquire into the experimental conditions that facilitate self-projection, for example, manipulating the personal relevance (D’Argembeau and Mathy, 2011; Cole and Berntsen, 2016; Cole et al., 2016) or familiarity (Robin and Moscovitch, 2014) of episodic cues, or even basic spatial attention processes proven capable to orient individuals in (past vs. future) time (Anelli et al., 2016), to embed them in more refined cued DD protocols.

## Data Availability Statement

The raw data supporting the conclusions of this article will be made available by the authors, without undue reservation, to any qualified researcher.

## Ethics Statement

The studies involving human participants were reviewed and approved by Bioethical Committee of the University of Bologna. The patients/participants provided their written informed consent to participate in this study.

## Author Contributions

EC, MS, and GP conceived and designed the research. MS and GT collected the data. EC and MS analyzed the data. All authors discussed the findings. EC and MS drafted the article. All authors revised it and approved its final version.

## Conflict of Interest

The authors declare that the research was conducted in the absence of any commercial or financial relationships that could be construed as a potential conflict of interest.
